# Bacterial Regulatory Proteins

**DOI:** 10.3390/ijms23126854

**Published:** 2022-06-20

**Authors:** Jan Kormanec

**Affiliations:** Institute of Molecular Biology, Slovak Academy of Sciences, 845 51 Bratislava, Slovakia; jan.kormanec@savba.sk; Tel.: +421-(2)-59307419

The regulation of gene expression in bacteria occurs predominantly at the level of transcription, which is controlled by RNA polymerase. The specificity of this process is ensured by sigma factors, which are essential regulatory subunits of RNA polymerase conferring promoter specificity. They allow the recognition of specific promoters, thereby controlling the expression of a specific set of genes (the so-called regulon of the corresponding sigma factor). Two unrelated families of sigma factors have been identified in bacteria. The dominant σ^70^ is present in all bacteria, and the minor σ^54^ is present only in few bacterial species. The σ^70^ family comprises several groups, including essential primary sigma factors controlling the expression of most housekeeping genes, non-essential homologues of primary sigma factors, alternative sigma factors, and distantly related extracytoplasmic function (ECF) sigma factors [1]. In addition to sigma factors, different families of regulatory proteins are involved in the regulation of gene expression at the transcriptional level. Negative regulators (repressors) usually bind to promoters and block the access of the RNA polymerase to initiate transcription. On the other hand, positive regulators (activators) usually bind in the upstream regions of promoters and allow efficient transcriptional activation upon association with RNA polymerase [2].

In this Special Issue, “Bacterial Regulatory Proteins”, a total of 14 interesting papers were published, consisting of ten original research papers and four reviews presenting new findings in this broad area of research.

Six research papers have described various aspects of regulation in streptomycetes. Bacteria of the genus *Streptomyces* are important producers of many biologically active secondary metabolites with a variety of biological activities, including antimicrobial, antifungal, antitumor, antiviral, anthelmintic, and immunosuppressive effects. They are saprophytic filamentous aerobic soil bacteria that undergo a complex process of morphological differentiation (involving three specific cell types: multinucleoid substrate mycelium, reproductive aerial mycelium, and unigenomic spores), which is accompanied by so-called “physiological differentiation”, which involves the production of various biologically active secondary metabolites [3].

The *Streptomyces* gene expression program is extremely complex. The genome of the best-studied *S. coelicolor* A3(2) strain has been shown to contain genes for 65 sigma factors of RNA polymerase. In contrast to unicellular *Bacillus subtilis*, which contains one general stress response sigma factor SigB regulated by a partner-switching phosphorylation mechanism with anti-sigma factor RsbW, anti-anti-sigma factor RsbV, and two PP2C phosphatases, *S. coelicolor* contains nine SigB homologues with a major role in differentiation and response to osmotic stress, 45 RsbW homologues, 17 RsbV homologues, and 44 activating PP2C phosphatases [4]. Sevcikova et al. [5] identified and characterized the promoters recognized by these nine SigB homologues and corresponding regulated genes (regulons) in *S. coelicolor* A3(2). All promoters showed high similarity in the -35 and -10 regions, and several of them were cross-recognized by multiple sigma factors. However, immunoblot analysis revealed the presence of several SigB homologues at different developmental stages, allowing the recognition of specific promoters and controlling the expression of the corresponding genes only at these developmental stages. These results suggest a complex regulation of the stress response in relation to morphological differentiation in *S. coelicolor* A3(2) by these sigma factors.

Fernández-García et al. [6] thoroughly investigated the novel regulatory protein SCO2102 in *S. coelicolor* A3(2). It contains a DnaA II protein–protein interaction domain, and the gene knockout phenotype suggests its essential role in morphological differentiation. It co-localized with the essential cell division protein FtsZ in the sporulating aerial hyphae, but it greatly reduced FtsZ-eGFP Z-ladder formation. SCO2102 positively affected the expression of the upstream gene *SCO2103* encoding methylenetetrahydrofolate reductase, which is involved in methionine and dTMP synthesis. Similar to *SCO2102*, the knockout of the *SCO2103* gene suggested its critical role in morphological differentiation. Both proteins also co-localize in the sporulating aerial hyphae, and the appearance of SCO2103 is dependent on SCO2102. These results suggest an interesting role for SCO2102 in positioning SCO2103 methylenetetrahydrofolate reductase in sporulating hyphae to facilitate nucleotide biosynthesis for chromosomal replication during the process of septation of aerial hyphae into unigenomic prespore compartments.

Two other papers reported research on novel regulatory proteins involved in the process of the regulation of physiological differentiation (production of bioactive secondary metabolites) in *Streptomyces*. Pawlik et al. [7] described the role of SCO3933, a novel transcription factor belonging to the GntR family, in the regulation of antibiotic production in *S. coelicolor* A3(2). It specifically bound to promoters in the actinorhodin and coelimycin biosynthetic gene clusters (BGCs), and its overproduction led to increased production of both antibiotics, suggesting its positive role in the regulation of both antibiotics in *S. coelicolor* A3(2). Interestingly, SCO3933 is located in a pSAM2-like insertion sequence in the genome. Therefore, these results also present an interesting regulatory link between integrative and conjugative elements and secondary metabolism.

Novakova et al. [8] described a new family of transcriptional co-activators activating secondary metabolism in several *Streptomyces* strains upon interaction with two different families of transcriptional activators. They differ by a small conserved C-terminal domain. The co-activators lacking this domain (represented by OvmZ from oviedomycin BGC) interact with a small positive regulator (represented by OvmW from oviedomycin BGC), whose gene is located downstream of the co-activator gene. On the other hand, larger co-activators (represented by Aur1O from auricin BGC) containing this conserved domain interact with transcriptional activators of the atypical response regulator (ARR) family (represented by Aur1P from auricin BGC).

The last two *Streptomyces* papers report strategies to activate silent BGCs by manipulating genes encoding regulatory proteins. Recent genome sequence data of approximately 1000 different *Streptomyces* strains revealed a large number of BGCs for unknown secondary metabolites (an average of 39 BGCs per genome). However, most of them are silent under laboratory conditions. Nevertheless, these *Streptomyces* BGCs represent a huge potential for the production of novel biologically active secondary metabolites [9].

Mingyar et al. [10] described a new strategy to activate these silent BGCs in several *Streptomyces* strains by the integration of foreign genes encoding positive transcriptional activators of different families under the control of the strong ermEp* promoter in these strains. Several already known antibiotics have been induced by this strategy, suggesting the usefulness of this strategy in identifying new potentially bioactive compounds.

González-Quiñónez et al. [11] reported a different strategy in this regard based on modulation of cytosolic copper, which is a modulator of differentiation and secondary metabolism in streptomycetes. The down-regulation of the *S. coelicolor* A3(2) operon *SCO2730/SCO2731*, which encodes the copper chaperone and copper export ATPase, in several *Streptomyces* strains using a newly created consensus antisense mRNA resulted in the activation of several BGCs, including some silent BGCs.

Four other research papers describe different aspects of the regulation of pathogenic bacteria. Bacterial pathogens causing various infections in humans have received much attention due to their great impact on human health. Many regulatory proteins, including sigma factors of RNA polymerase, have been reported to play a critical role in the pathogenicity of various bacterial pathogens [1].

Elhawy et al. [12] characterized PtpB arginine phosphatase in the *Stahylococcus aureus* regulatory network to evade innate host immunity. This versatile opportunistic human pathogen causes a variety of diseases, ranging from superficial skin infections to life-threatening illnesses such as bacteremia, sepsis, endocarditis, osteomyelitis, and toxic shock syndrome. PtpB modulates the arginine phosphorylation states of several response regulators, and the deletion of its gene reduces survival in macrophages, suggesting its role in pathogenity. PtpB affected the expression of several genes or operons encoding virulence factors— *aur* (encoding the zinc-metalloprotease aureolysin), *nuc* (encoding thermonuclease), and *psm*α (encoding modulins α1–4)*—*and also the expression of small regulatory RNA *RNAIII*, one of the major regulators controlling exoprotein synthesis in *S. aureus*. In addition, the deletion of *ptpB* in *S. aureus* SA564 dramatically reduced the mutant’s ability to degrade extracellular DNA, hydrolyze extracellular proteins, and resist Triton X-100 induced autolysis. This deletion also impedes the ability of *S. aureus* to avoid phagocytosis by polymorphonuclear leukocytes. All of these results suggest that PtpB contributes positively to the ability of *S. aureus* to evade host innate immunity.

Bukowski et al. [13] characterized a novel transcription factor SaoC and its antagonist SaoB in *S. aureus.* Both genes are located in the *saoABC* operon, which is specific to the genus *Staphylococcus* and encodes three annotated hypothetical proteins. However, SaoC contains a DNA binding domain at its N-terminus. The deletion of all three genes in *S. aureus* revealed that two of them affect the physiology and pathogenicity of the bacterium. Both *S. aureus* Δ*saoB* and Δ*saoC* mutants negatively affect growth in the minimal medium, and the Δ*saoC* mutant negatively affects intracellular survival in human fibroblasts, whereas the Δ*saoB* mutant produces a 4-fold increased number of persisters. Comparative RNAseq analysis in the wild type and the Δ*saoC* mutant revealed that SaoC affects the expression of genes involved in amino acid transport and metabolism, and several virulence factor genes. These results suggest that this novel transcription factor SaoC links the metabolic state of cells to manifestations of virulence, with SaoB acting as its proposed antagonist.

Benthien et al. [14] investigated a role of the transcription factor SpoVG in biofilm formation in *Staphylococcus epidermidis*, a common cause of infections related to medical devices. The deletion of the *spoVG* gene resulted in a failure of biofilm formation under in vitro conditions, clearly indicating a critical role for this transcription factor in this process. Consistent with this hypothesis, the *spoVG* mutant affected the expression of the intercellular adhesion (*ica*) locus responsible for biofilm formation by down-regulating the *icaADBC* expression and up-regulating the *icaR* operon repressor gene. These data were confirmed by binding of SpoVG to the respective promoter regions. However, the *spoVG* mutant hardly affected biofilm formation in an in vivo mouse model of infection. Although all these results clearly indicate a critical role of SpoVG in biofilm formation of *S. epidermis* under in vitro conditions, it appears to be dispensable for this process under in vivo conditions.

Zannoni et al. [15] investigated different aspects of the interaction between the essential response regulator HP1043 of the bacterial two component systems and its cognate *Php1227* promoter in *Helicobacter pylori.* This widespread human pathogen causes several gastrointestinal diseases. These studies identified nucleotides of the promoter consensus sequence and amino acid residues in the DNA binding domain of HP1043 that are essential for binding. Interestingly, unlike most other response regulators of the OmpR/PhoB family, structural modeling suggests that HP1043 binds to the *Php1227* promoter in a head-to-head conformation.

The last four articles in this issue are reviews that summarize several interesting families of sigma factors of RNA polymerase and various mechanisms of bacterial regulatory proteins in stress response and other cellular processes. Marcos-Torres et al. [16] comprehensively updated the different regulatory mechanisms activating non-canonical extracytoplasmic function (ECF) sigma factors of RNA polymerase. Unlike most canonical ECF sigma factors, which are sequestered by membrane-bound anti-sigma factors, this interaction is released by several extracellular or intracellular stimuli, and different mechanisms have been described for other non-canonical ECF sigma factors, e.g., partner-switching, phosphorylation-dependent mechanisms, and metal-sensing.

Yu et al. [17] summarized the various physiological roles of sigma factors of the σ^54^ family in phytopathogenic bacteria. σ^54^ is distinct from all other sigma factors. Unlike sigma factors of the σ^70^ family, σ^54^ is unable to form an open complex upon association with RNA polymerase. This step requires the assistance of transcriptional activators (enhancer-binding proteins), which utilize ATP hydrolysis to perform the conformational changes necessary for this transition. In different bacteria, σ^54^ regulates various biological processes, e.g., nitrogen metabolism, flagella synthesis, motility, antibiotic resistance, and virulence. This review summarizes the current knowledge on various regulatory functions of σ^54^, including bacterial growth, motility, flagella synthesis, type III secretion system, and virulence-associated phenotypes in various phytopathogenic bacteria (e.g., *Pseudomonas syringae*, *Ralstonia solanacearum*, *Xanthomonas* spp., *Erwinia amylovora*, and *Dickeya dadantii*).

Pis Diez et al. [18] summarized the current knowledge on the mechanisms of bacterial transcriptional regulators to cope with different environmental stimuli. In their natural habitats, bacteria are exposed to various stresses. The perception of stress stimuli and responses are mediated by various bacterial transcriptional factors that induce expression genes necessary to overcome these adverse conditions. Authors discussed recent research results on allosteric sensing mechanisms and the regulation of transcriptional regulators, focusing on multi-target strategies and novel experimental techniques. They comprehensively described the state-of-the-art techniques used for the functional, biophysical, structural, and dynamic characterization of different types of allosteric bacterial regulatory proteins. In addition, they provided a roadmap that can be used for experimental design in the study of bacterial regulatory systems and information on databases containing available structural information on particular families of transcriptional regulators.

In the last review, Jannette Carey [19] addressed the evolution of our current understanding of the molecular origins of affinity and specificity in the interactions of bacterial regulatory proteins with DNA. Both parameters are crucially responsible for the correct expression of the directed genes and consequently for very precise levels of the encoded proteins that ensure their precise function in the life processes of the cell.
